# Age at First Use of Alcohol and Risk of Heavy Alcohol Use: A Population-Based Study

**DOI:** 10.1155/2013/721761

**Published:** 2013-12-29

**Authors:** Wenbin Liang, Tanya Chikritzhs

**Affiliations:** National Drug Research Institute, Health Science, Curtin University, GPO Box U1987, Perth WA 6845, Australia

## Abstract

*Aim*. To examine the association between age at first alcohol use and risk of heavy alcohol use among the adult US general drinking population. *Methods*. This population-based study used the 2010 National Survey on Drug Use and Health (NSDUH) from United States. Multivariate Poisson regression was employed to predict the frequency of heavy alcohol use (five or more drinks per occasion) in the last 30 days with age at first use of alcohol controlling for potential confounding factors. *Results*. Younger age at first use of alcohol was associated with increased likelihood of heavy alcohol use in the last 30 days in this population-based sample. This association remained significant when analysis was reperformed for the subgroup of participants who were with desired good health status and Kessler score lower than 12. *Conclusion*. Younger age at first use of alcohol was associated with increased likelihood of heavy alcohol use.

## 1. Introduction

Several studies have suggested that people who start drinking at a younger age have an increased risk of alcohol use disorders [1–6]. It has been hypothesized that the interactions among genetic and environmental factors play an important role in this observed association; however, the underlying causal mechanism is not fully understood [6]. Our recent analysis of data collected by the Australian National Drug Strategy Household Survey showed that age at first use was associated with a higher level of subsequent alcohol use among the general Australian population [7]. This association remained significant even after the analysis was limited to the population who had good (i.e., “desired”) health status and low psychological distress [7]. To determine whether this observation is specific to the Australian population or generalizable, it is important to further investigate the relationship between early onset of alcohol use and heavy alcohol use in other populations. If early onset of alcohol use independently (i.e., independent of other socioeconomic and environmental risk factors) leads to heavy drinking behaviours, then it may be postulated that, to some extent, early alcohol use causes alcohol use disorders by increasing the frequency of heavy alcohol use. In this study, we aimed to examine the association between age at first alcohol use and risk of heavy alcohol use among the adult US general drinking population.

## 2. Method

Details of sampling strategy and data collection methods have been described in the 2010 National Survey on Drug Use and Health (NSDUH) report [8, 9]. Briefly, the NSDUH is a national survey targeting the 12-year and older noninstitutionalized population of the United States. The 2010 survey used computer-assisted face-to-face interviews to collect drug use information and demographic information from respondents. In order to produce both national and state level estimates, multistage stratification and randomization combined sampling method was used [8]. In the 2010 NSDUH, participants were asked detailed questions about their alcohol use, including age at first use of alcohol. The original question was: “Think about the first time you had a drink of an alcoholic beverage. How old were you the first time you had a drink of an alcoholic beverage? Please do not include any time when you only had a sip or two from a drink.” In the 2010 NSDUH, a “drink” was defined as “a can or bottle of beer, a glass of wine or a wine cooler, a shot of liquor, or a mixed drink with liquor in it.” Participants were also asked about the frequency of heavy alcohol use over the last month. Heavy alcohol use was defined as consuming 5 or more drinks on the same occasion. The original question asked in the survey was: “During the past 30 days, that is since (the date 30 days before the interview), on how many days did you have 5 or more drinks on the same occasion? By occasion, we mean at the same time or within a couple of hours of each other” [8]. In this study, we investigated whether age at first use of alcohol predicted frequency of heavy alcohol (in the last 30 days) at older ages.

### 2.1. Data Analysis

Multivariate Poisson regression was employed to predict the frequency of heavy alcohol use (more than 5 drinks per occasion) in the last 30 days with age at first use of alcohol. Potential confounding factors, including current age, gender, level of achieved education, income, marital status, general health status, and psychological distress level in the last 30 days, were controlled in the model. Sampling weight provided in the dataset was applied in the analysis. The analysis was first performed with all adult participants (age ≥18) who consumed alcohol in the last 12 months. There were 29,315 participants aged 18 years at the time of the survey and who had consumed alcohol in the last twelve months. Less than 3% of these participants did not provide necessary information on the dependent variables or some of the independent variables and therefore were excluded from the multivariate analysis. Given that heavy alcohol use was more prevalent among young adults, the analysis was independently repeated for those aged 18–29 years and 30–49 years.

As noted in our previous study using an Australian sample, physical and mental morbidities may influence the relationship between age at first use of alcohol and frequency of heavy alcohol use [7]. The 2010 NSDUH included the Kessler 6 scale and a question on general health [8]. The Kessler 6 scale is a sensitive measure of the existence of nonspecific mental conditions. A score of 0–12 indicates low risk of a mental health condition being present [10–12]. Self-reported health status with five categories (excellent, very good, good, fair, and poor) was used as the indicator of health in the survey. Self-reported health status has also been shown to be a valid and reliable measurement of general health and has been repeatedly used in the NSDUH and other national surveys [13–15]. Limiting the sample to those with a low Kessler score and good self-rated health status substantially reduces the likelihood that any observed association will be influenced by the presence of physical or mental disorders [7, 14, 16–18]. We therefore repeated the analysis on a subsample limited to those with Kessler scores of 12 or less and with a self-rating of health that was good, very good, or excellent.

In order to further control for residual confounding effects, a proxy outcome (number of days smoked in the last 30 days) was used to estimate the effects of residual confounding effects. A similar approach has been used to examine the “protective” effect of moderate alcohol use [16, 17]. This was achieved by using number of days smoked in the last 30 days as the outcome variable instead of frequency of heavy alcohol use in the last 30 days, while keeping all the controlled variables unchanged. Participants included those aged 18 years at the time of the survey who had consumed alcohol in the last twelve months. This approach operates on the basis that alcohol use and tobacco use share a similar set of confounders [19–23]. We also assume that any causal relationship between age at first use of alcohol and tobacco at older ages is weaker than age at first use of alcohol and future heavy alcohol use. Any observed effects of age at first use on tobacco use were thus controlled for in the final model.

## 3. Results

From the univariate analysis, it was observed that the prevalence of consuming more than 5 drinks on the same occasion at least once in the last 30 days was significantly higher among participants who started consuming alcohol before the age of 18 years among both males and females. Similar results were observed when analyses were limited to those between 20 and 29 years and those between 30 and 49 years (Table 1). The multivariate analysis also indicated that starting to drink alcohol at a younger age was associated with significantly increased risk of heavy alcohol use over the last 30 days. Participants with less desirable health status or with a Kessler 6 score higher than 12 were significantly more likely to engage in heavy alcohol use in the last 30 days. Participants who were 65 years or older were least likely to engage in heavy alcohol use (Table 2). Among the subgroup of participants aged 20–29 years who rated their health as good, very good, or excellent and who had a Kessler score lower than 12, having consumed alcohol before the age of 18 years remained a significant risk factor for heavy alcohol use. Conversely, having first consumed alcohol at or after the age of 25 years reduced the risk of heavy alcohol use. Similar results were observed for those aged 30–49 years (Table 3).

Significant associations between age at first use of alcohol and smoking were observed. The adjusted relative risks for smoking were 1.49 (95% confidence interval: 1.29, 1.70), 1.57 (1.43, 1.72), 1.36 (1.25, 1.49), and 1.00 (reference level) for first use of alcohol before 12 years, 12–14 years, 15–17 years, and 18–24 years, respectively. After controlling for these effects, the relative risk of heavy alcohol use by first use of alcohol remained significant, although the effect size was somewhat reduced to 2.03 (1.68, 2.45), 1.66 (1.46, 1.89), 1.41 (1.25, 1.59), and 1.00 (reference) for first use of alcohol before 12 years, 12–14 years, 15–17 years, and 18–24 years, respectively.

## 4. Discussion

In this study, we investigated the association between age at first use of alcohol and current alcohol consumption using US data from the 2010 National Survey on Drug Use and Health. Younger age at first use of alcohol was associated with increased likelihood of heavy alcohol use in the last 30 days in this population-based sample. This association was not dependent on level of psychological distress and health status. The findings of this study are consistent with the findings from a previous study of Australia's population [7]. This also suggests that the association between early onset of alcohol use and heavy alcohol consumption in later life cannot be fully explained by concurrent negative socioeconomic and environmental factors that usually influence the risk of physical diseases and mental disorders including alcohol use disorders [14, 18, 24, 25].

The causal mechanism underlying this observed association is not fully understood. It is plausible that starting to use alcohol before the age of 18 years directly increases the risk of developing heavy alcohol use behaviours. The risk of developing habitual heavy alcohol use before the age of 18 years is nil for a person who starts drinking after the age of 18 years. However, in keeping with the relatively high prevalence of frequent heavy alcohol use among adolescents, the risk well exceeds zero for a person who starts drinking before the age of 18 years [26, 27]. In addition, compared to adults, adolescents are more likely to engage in sensation-seeking and risk-taking behaviours [9, 16]; therefore given access to alcohol, the risk of frequent heavy alcohol use may be higher in adolescence than in adulthood. It is nevertheless important that parental attitudes and behaviours during their children's initial exposure to alcohol may have substantial effects on their future consumption patterns [28–32].

The causal relationship between heavy alcohol use and alcohol use disorders is clear as heavy alcohol use is a necessary condition for the development of any alcohol use disorder; it follows therefore that increased risk of heavy alcohol use will further increase the risk of alcohol use disorders. Onset of alcohol use before the age of 18 years would therefore increase the risk of alcohol use disorders. Abstinence from alcohol until the age of 18 years will reduce the risk of alcohol-related harm. These findings are closely relevant to legislations on minimum drinking age.

## 5. Conclusion

Younger age at first use of alcohol was associated with increased likelihood of heavy alcohol use.

## Figures and Tables

**Table 1:** Prevalence of heavy alcohol use in the last 30 days by age at first use of alcohol.

Age at first use of alcohol	Male			Female		
	Heavy alcohol use over the last 30 days			Heavy alcohol use over the last 30 days		
	No	Yes	% Yes	No	Yes	% Yes
Age group ≥ 18						
<12	275	510	65%	218	196	47%
12–14	949	1866	66%	1402	1315	48%
15–17	2543	3431	57%	3725	2363	39%
18–24	2427	1667	41%	4084	1179	22%
≥25	98	37	27%	271	45	14%

Age group 18–29						
<12	126	314	71%	121	137	53%
12–14	459	1229	73%	771	983	56%
15–17	1281	2350	65%	1941	1794	48%
18–24	1357	1099	45%	2066	788	28%
≥25	13	3	19%	8	1	11%

Age group 30–49						
<12	103	159	61%	72	53	42%
12–14	360	527	59%	518	303	37%
15–17	856	859	50%	1341	479	26%
18–24	641	439	41%	1232	300	20%
≥25	60	24	29%	155	27	15%

**Table 2:** Estimates from Poisson regression model.

	IRR	(95% conf. interval)
Gender		
Male	1.00	
Female	0.42	0.38–0.46
Age at first use of alcohol		
<12	3.02	2.50–3.65
12–14	2.61	2.30–2.97
15–17	1.93	1.71–2.17
18–24	1.00	
≥25	1.44	0.77–2.69
Current age		
18-19	1.00	
20–25	1.57	1.42–1.73
26–29	1.35	1.17–1.55
30–34	1.39	1.19–1.64
35–49	1.23	1.06–1.41
50–64	1.02	0.84–1.24
65+	0.60	0.42–0.86
Highest education level attained		
Less than high school	1.00	
High school graduate	1.04	0.90–1.19
Some college	0.91	0.78–1.05
College graduate or above	0.80	0.68–0.93
Total family income		
<$20,000	1.00	
$20,000–$49,999	0.85	0.76–0.95
$50,000–$74,999	0.89	0.77–1.03
$75,000 or More	0.82	0.72–0.93
Self-rated health status		
Excellent	1.00	
Very good	1.03	0.93–1.14
Good	1.13	1.00–1.27
Fair	1.40	1.15–1.70
Poor	1.32	0.90–1.92
Kessler score		
≤12	1.00	
≥13	1.27	1.08–1.48
Marital status		
Married	1.00	
Widowed	1.01	0.68–1.49
Divorced or separated	1.47	1.25–1.73
Never been married	1.50	1.34–1.68
Language used in the survey		
English	1.00	
Spanish	0.64	0.50–0.81

IRR: incidence rate ratios.

**Table 3:** Association between age at first use of alcohol and likelihood of heavy alcohol use in the last 30 days among subsample with low Kessler score and good, very good, or excellent self-rated physical health.

Age at first use of alcohol	IRR	(95% conf. interval)	IRR*	(95% conf. interval)
Age group 18–29				
<12	3.30	2.74–3.98	3.19	2.62–3.88
12–14	3.36	2.98–3.78	3.35	2.95–3.80
15–17	2.44	2.19–2.72	2.47	2.20–2.76
18–24	1.00		1.00	
≥25	0.18	0.06–0.51	0.19	0.06–0.55

Age group 30–49				
<12	2.66	2.04–3.45	2.54	1.89–3.42
12–14	2.06	1.68–2.53	2.03	1.63–2.52
15–17	1.60	1.32–1.94	1.60	1.30–1.96
18–24	1.00		1.00	
≥25	0.40	0.26–0.61	0.43	0.27–0.68

IRR: incidence rate ratios.

Models controlled for all variables showed in Table 2.

*Estimations from subsample with Kessler scores of 12 or less and a self-rating of health as good, very good, or excellent.
